# Retraction Note: MNAT1 is overexpressed in colorectal cancer and mediates p53 ubiquitin-degradation to promote colorectal cancer malignance

**DOI:** 10.1186/s13046-021-02204-1

**Published:** 2021-12-24

**Authors:** Shan Zhou, Jinping Lu, Yuejin Li, Chan Chen, Yongqiang Cai, Gongjun Tan, Zhengke Peng, Zhenlin Zhang, Zigang Dong, Tiebang Kang, Faqing Tang

**Affiliations:** 1 Department of Clinical Laboratory, Hunan Cancer Hospital & The Affiliated Cancer Hospital of Xiangya School of Medicine, Central South University, Changsha, 410013 China; 2 Department of Clinical Laboratory, Zhuhai Hospital, Jinan University, Zhuhai, 519000 Guangdong China; 3 Hormel Institute, University of Minnesota, 801 16th Avenue NE, Austin, MN 55912 USA; 4 State Key Laboratory of Oncology in South China and Department of Experimental Research, Sun Yat-sen University Cancer Center, Guangzhou, 510060 Guangdong China

**Retraction Note: J Exp Clin Cancer Res 37, 284 (2018)**

**https://doi.org/10.1186/s13046-018-0956-3**

The Editor-in-Chief has retracted this article at the request of the authors. After publication, concerns were raised about the data presented in Fig. 8c having been published elsewhere representing different samples [1–5]. The authors checked their data and noticed that inaccurate results were presented in Fig. 1B, 3F and 5E. Further investigation revealed the following additional issues:

- In Fig. 4D, the background of parts of the Flag bands appears completely smooth, as if parts of the image have been removed.
- In Fig. 5E (right panel), there is a noticeable break in the background of the blot, and the two parts are misaligned in the image.
- In Fig. 6C,
  - ○ the image of the bands presented for MNAT1 (LoVo cells) appears highly similar to that in Fig. 2D DLD1 cells;
  - ○ MDM2 and MNAT1 and HSP60 (HEK293T cells) appear highly similar to Fig. 2A MNAT1 and GAPDH (HCT116 cells);
  - ○ p53 (LoVo cells) bands apprat highly similar to those presented for MNAT1 in Fig. 6D.
- In Fig. 7A, the GAPDH bands appear highly similar to the HSP70 bands in Fig. 3A-b.

The data reported in this article are therefore unreliable. In addition, the authors were unable to provide a copy of the original Ethics Approval for the animal study upon the Editor's request, which raises further concerns about the integrity of the article.

None of the authors have responded to any correspondence from the editor or publisher about this retraction.
